# New Promising Targets for Synthetic Omptin-Based Peptide Vaccine against Gram-Negative Pathogens

**DOI:** 10.3390/vaccines7020036

**Published:** 2019-04-10

**Authors:** Valentina A. Feodorova, Anna M. Lyapina, Sergey S. Zaitsev, Maria A. Khizhnyakova, Lidiya V. Sayapina, Onega V. Ulianova, Sergey S. Ulyanov, Vladimir L. Motin

**Affiliations:** 1 Laboratory for Molecular Biology and NanoBiotechnology, Federal Research Center for Virology and Microbiology, Branch in Saratov, 410028 Saratov, Russia; lyapina_anna@inbox.ru (A.M.L.); zaytsev-sergey@inbox.ru (S.S.Z.); khizhnyakova_mariya@mail.ru (M.A.K.); ulianovaov@mail.ru (O.V.U.); 2 Department of Vaccine Control, Scientific Center on Expertise of Medical Application Products, 127051 Moscow, Russia; l.v.sayapina@mail.ru; 3 Department for Medical Optics, Saratov State University, 410012 Saratov, Russia; prof.sergey.ulyanov@outlook.com; 4 Department of Pathology, Department of Microbiology and Immunology, University of Texas Medical Branch, Galveston, TX 77555, USA

**Keywords:** omptin family proteases, Gram-negative pathogens, peptide ELISA, B-cell epitope, peptide vaccine, broad-spectrum vaccine, multi-pathogen vaccine, vaccine development

## Abstract

Omptins represent a family of proteases commonly found in various Gram-negative pathogens. These proteins play an important role in host–pathogen interaction and have been recognized as key virulence factors, highlighting the possibility of developing an omptin-based broad-spectrum vaccine. The prototypical omptin, His-tagged recombinant Pla, was used as a model target antigen. In total, 46 linear and 24 conformational epitopes for the omptin family were predicted by the use of ElliPro service. Among these we selected highly conserved, antigenic, non-allergenic, and immunogenic B-cell epitopes. Five epitopes (2, 6, 8, 10, and 11 corresponding to Pla regions 52–60, 146–150, 231–234, 286–295, and 306–311, respectively) could be the first choice for the development of the new generation of target-peptide-based vaccine against plague. The partial residues of omptin epitopes 6, 8, and 10 (regions 136–145, 227–230, and 274–285) could be promising targets for the multi-pathogen vaccine against a group of enterobacterial infections. The comparative analysis and 3D modeling of amino acid sequences of several omptin family proteases, such as Pla (*Yersinia pestis*), PgtE (*Salmonella enterica*), SopA (*Shigella flexneri*), OmpT, and OmpP (*Escherichia coli*), confirmed their high cross-homology with respect to the identified epitope clusters and possible involvement of individual epitopes in host–pathogen interaction.

## 1. Introduction

Currently, infectious diseases (IDs) are responsible for about 30% of global human mortality per year [1,2]. They are recognized as a significant burden on the public health and economic stability of societies all over the world [3]. During the course of human history, IDs have been accountable for a number of devastating epidemics that have regularly threatened the evolution and even the existence of human civilization [4]. Annually, emerging and neglected IDs affect more than one billion people in more than 149 countries, including chronically infected individuals [5,6]. For instance, diarrheal IDs are the second leading cause of death in children under five years old, and are responsible for killing around 525,000 children every year. Globally, there are nearly 1.7 billion cases of childhood diarrheal IDs every year [7].

It is considered that vaccines have achieved extraordinary success in the history of public health, yielding substantial reductions in worldwide morbidity and mortality from IDs over the past half century [8,9]. This has been proven by the accomplishment of smallpox eradication, the drastic reduction in polio cases over the past 20 years, the progress toward tetanus elimination, and the reduction in measles mortality [10]. Vaccination of at-risk populations against plague, tularemia, brucellosis, and a number of other emerging and neglected IDs has been the most effective way to significantly improve the control over disease and deaths from these IDs globally [10,11]. Successful efforts to develop vaccines against acute diarrhea IDs over more than 40 years have resulted in a certain decline in childhood mortality in the past decades [12,13,14].

Among a number of different types of vaccines, the WHO recognizes four main types, categorized by the antigen used in their preparation: (i) live attenuated (LAV); (ii) inactivated (killed antigen); (iii) subunit (purified antigen); and (iv) toxoid (inactivated toxins). Further, vaccines may be classified as: (i) monovalent, containing a single antigen to protect against a particular ID); (ii) polyvalent, containing two or more variants of the same antigen aiming to protect against a distinct ID; and (iii) combination vaccine, consisting of several individual specific antigens combined in a single injection to prevent the relevant target diseases or to protect against multiple strains of infectious agents causing the same disease [15]. Unfortunately, vaccines based on a single antigen often fail to induce immunity against a group of IDs. In fact, the majority of existing vaccines are carefully targeted at an individual specific antigen substance and are protective only against a single related infectious disease [10,14,16]. Moreover, the protective effect of these vaccines is based on duplicating the immune responses induced by natural infections [17]. Recent advances in immunology, structural biology, and synthetic chemistry have heralded the unprecedented rational development of a new generation of effective and safe vaccines that will hopefully be instrumental in controlling many IDs [17,18,19].

A number of pathogens enter the body via mucosal surfaces using airways, intestines, etc., overcoming innate immunity to establish the infection. This highlights the necessity to induce an efficient mucosal immune response, which is critically important for protection against IDs caused by both foodborne (enteric) and airborne pathogens. Th1 and Th17 cell-mediated immune responses play a critical role in the induction of accelerated and efficient mucosal immune responses. Moreover, the induction of Th17 polarization elicits a protective effect directed to outer membrane proteins (OMPs), providing clade-specific serovar-independent immunity including encapsulated bacteria [20]. Thus, the identification of specific antigens capable of inducing a robust Th17 response to the common prevalent pathogens is one of the critical steps in effective vaccination strategy [21]. 

Understanding the epitope–antibody interaction is the key for the construction of potent vaccines. B-cell epitope mapping is a promising approach for identifying the main antigenic determinants of microorganisms. Epitope-based vaccines have a remarkable advantage over the conventional ones, since they are safe, strictly specific, able to avoid undesirable immune responses, generate long-lasting immunity, and are reasonably cheaper [22]. In fact, the B-cell epitope mapping of individual target antigen(s) can aid in the identification of both strictly specific and broadly-specific immunodominant epitopes that facilitate the development of both mono-peptide and multi-peptide vaccines against either a single or several IDs. Here we attempted to identify promising molecular targets in the form of linear immunoreactive epitopes suitable for the future development of a new generation of an effective synthetic vaccine against a group of IDs, caused by several Gram-negative pathogens, namely *Salmonella*, *Shigella*, *Escherichia coli*, and *Yersinia pestis*. These microbes infect both humans and animals and are associated with high levels of morbidity and mortality, an increased emergence of multidrug-resistant strains, as well as with the need to cover protection against different serovars [23].

For this purpose, we investigated the omptins family of outer membrane proteases commonly found in these pathogens that are also known as omptin-expressing bacterial species [24]. These proteins play an important role in host–pathogen interaction and have been recognized as key virulence factors, highlighting the omptins as potential targets for antimicrobial drug and vaccine development [24,25,26]. The omptin members demonstrate a high level of protein homology (up to 75%), and have nearly identical 3D structures [25]. However, the location of their common immune reactive epitopes has not been elucidated thus far. This study intended to fill in this gap. We used the prototypical omptin as a model target antigen—a His-tagged recombinant Pla (pro-omptin) purified under denaturing conditions [27]. We screened a set of sera from human volunteers who were multiply vaccinated with live plague vaccine (LPV) to identify Pla-specific epitopes [28,29,30]. To reveal common anti-omptin epitopes, we also used a panel of sera from unvaccinated donors with detectable level of antibodies to Pla who had possible history of prior enteric bacterial diseases. Further, we used a library of 61 overlapping synthetic peptides to map B-cell immune-reactive epitopes to the pro-omptin by peptide ELISA. Finally, we predicted, identified, and proved 11 potential immune-reactive epitopes that are commonly presented in different omptins and could define omptin-mediated host–pathogen interaction. The identification of these commonly-shared immune-reactive epitopes will be helpful in the development of a new generation of effective synthetic omptin-based broad-spectrum peptide vaccine against a number of Gram-negative pathogens.

## 2. Material and Methods

### 2.1. Study Design

A detailed protocol of this study has been published, describing the study design, study population, and methods including consenting procedures, assessment of immune-serological B-cells, and T-cells immune responses of human donors vaccinated with LPV as well as non-vaccinated donors [28,29,30]. Overall, sera from vaccinated donors (*n* = 18) who received multiple annual immunizations with LPV and unvaccinated healthy individuals (*n* = 6) who had never been in contact with either *Y. pestis* or its antigens participated in the study. Unvaccinated donors including those who had an anamnestic immune response proved their possible previous history of enteric bacterial infections.

The study was conducted in compliance with the Declaration of Helsinki and the regulatory requirements of the Russian Federation. Study protocols were approved by the Institutional Review Board of The Saratov State Medical University named after V. I. Razumovsky (IRB Registration #: IRB00005197, Federal Wide Assurance (FWA) #: FWA00009567). Signed written informed consent was obtained from all participants.

### 2.2. Omptin Sequences

The sequences of the omptin proteins, including Pla (*Y. pestis* CO92), OmpT and OmpP (*E. coli*), PgtE (*Salmonella enterica*), and SopA (*Shigella flexneri*) were downloaded from GenBank (https://www.ncbi.nlm.nih.gov/genbank/). The sequence retrieval accession numbers for each individual omptin along with other information are listed in Table 1.

### 2.3. Prediction of Linear and Conformational B-Cell Epitopes

B-cell epitopes were predicted using ElliPro (http://tools.immuneepitope.org/toolsElliPro/) using both protein sequences and structural information for each omptin.

Ribbon diagram models of the three-dimensional (3D) structures of the omptin protein family were generated with Swiss-PdbViewer/DeepView v. 4.1 (https://spdbv.vital-it.ch/) and rendered in POV-Ray v. 3.7. (http://www.povray.org/), based on alignments of the amino acid sequences of Pla, OmpP, OmpT, PgtE, and SopA.

Ribbon diagram models of the 3D structures of the individual predicted immune-reactive omptin protein linear and conformational epitopes were generated by the ElliPro service.

### 2.4. Peptide Library Characteristics

A library of 61 overlapping synthetic peptides, each 15 amino acid residues in length (offset by 5 residues at a time) was made by GenScript, Piscataway, NJ, USA as described recently [30].

### 2.5. Predicted Antigenic and Allergenic Characteristics of the Omptin’s B-cell Epitopes 

AlgPred [31] (http://crdd.osdd.net), AllerTOP v. 2.0 (http://www.ddg-pharmfac.net/AllerTOP), and ANTIGENpro [32] (http://scratch.proteomics.ics.uci.edu/) services were used for prediction of allergenicity (AlgPred and AllerTop) and antigenicity (ANTIGEN).

## 3. Results

### 3.1. Omptin B-Cell Epitopes Prediction

**Figure 1:**
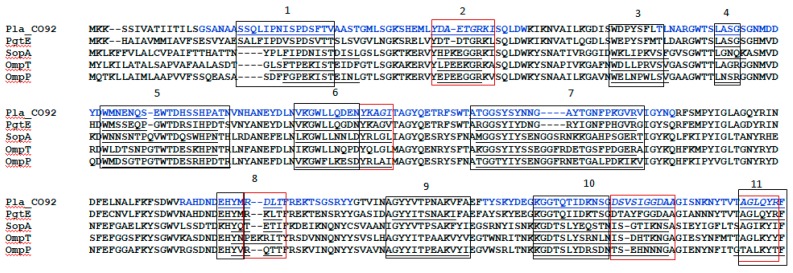
Identification of four major reactive epitopes (indicated in blue) within the pro-omptin molecule and their homology with other omptin family proteases. Alignment of the omptins of different organisms: Pla—*Yersinia pestis*, PgtE—*Salmonella enterica*, SopA—*Shigella flexneri*, OmpT and OmpP—*Escherichia coli*. Boxes indicate positions of immunodominant regions mapped via immunoreactive peptides.

Overall, ElliPro predicted 46 linear and 24 conformational epitopes for the omptin family (Table 1). The subsequent comparative analysis of the sequences of the pro-omptin Pla with other omptin family proteases, such as PgtE (*Salmonella enterica*), SopA (Shigella *flexneri*), and OmpT and OmpP (*E. coli*) revealed the location of predicted linear B-cell epitopes in either identical positions or in a very close proximity to all nine Pla epitopes predicted by ElliPro and identified serologically using human anti-Pla antisera [30]. Linear epitopes were numbered sequentially from 1 to 11 through the selected proteins of the omptin family. There were at least six linear epitopes common for all five omptins, namely epitopes 1, 2, 5, 7, 8, and 10: four (linear epitopes 4 and 6) for four omptins (omitted in only OmpT); three (linear epitopes 3 and 11) in omptins OmpP, OmpT, and SopA; and epitope 9 in only OmpP and PgtE (Figure 1). The omptin conformational epitopes were formed by the residues of all the relevant predicted linear epitopes for each of the omptins. Additionally, conformational epitopes may include some peptides located at the positions corresponding to one of the 11 omptin linear epitopes that were not predicted for the individual omptin protein. For instance, epitopes 3 and 9 were absent in Pla and were predictable for other omptins, such as OmpP, OmpT, and SopA (epitope 3) and OmpP and PgtE (epitope 9).

### 3.2. B-Cell Epitope Mapping with Human Sera

The predicted immuno-reactivity potency of the B-cell linear epitopes were tested and validated by their mapping with the use of a panel of human sera positive in ELISA and immunoblotting for the presence of antibodies to Pla at a detectable level [28,29,30]. For this purpose a library of 61 overlapping synthetic peptides designed on the basis of the amino acid sequence of the Pla pro-omptin was probed. The initial screening of the peptide library revealed a specific positive reaction of the representative sera samples with 35 out of 61 peptides (Table 2). 

These immuno-reactive peptides were grouped into four separate clusters of peptides, suggesting the presence of four major reactive regions within the pro-omptin molecule (Figure 1). These immuno-reactive regions included all nine individual linear B-cell epitopes predicted by using the ElliPro for Pla antigen. Each peptide cluster was formed by several overlapping peptides and occupied either a single or multiple (up to 2–3) separate epitopes. In fact, this provided summarized positive reactions with several different human sera into regions longer that a single predicted epitope. This means that cumulative positive reactions combined from several different human sera reacted with the same epitope formed by several overlapping peptides. No positive reaction was seen with the remaining two epitopes, which were not predicted for Pla while they were found in other omptins (epitopes 3 and 9). Importantly, there was 100% identity of the in silico predicted results with data obtained from the peptide ELISA (Table 1 and Table 2, Figure 1).

**Figure 2:**
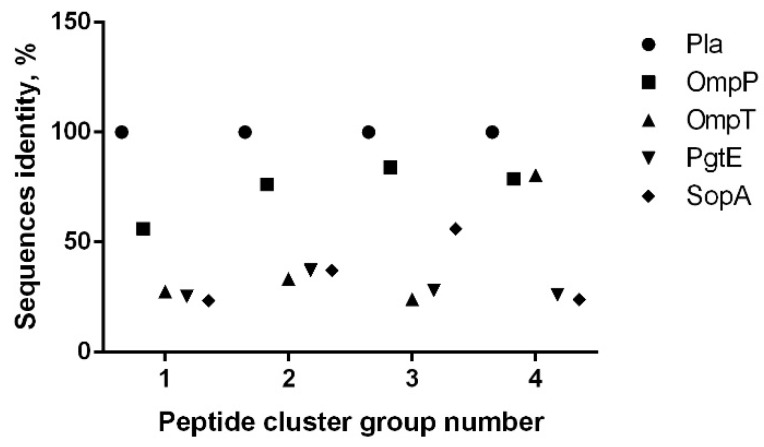
Identification of four major reactive epitopes within the pro-omptin molecule and their homology with other omptin family proteases. Percent homology between amino acid sequences of Pla versus other omptins in four immunodominant regions mapped via immunoreactive peptides.

The subsequent comparative analysis of the sequences of these four clusters of the pro-omptin Pla with other omptin family proteases revealed their cross-homology in the range of 26.6%–84.0% (Figure 2). The highest level of homology (56.0%%–84.0%) was observed with the OmpP protease within clusters 1–4. The cross-homology with the pro-omptin was at the level of 23.4%–80.0%. 

The epitopes specific for either Pla alone or the remaining four omptins were selected based on the positive reaction with human sera derived from either vaccinated or unvaccinated donors. Thus, in total there were five epitopes and certain regions of the epitopes specific for Pla only (epitopes 2, 6, 8, 10, and 11) and eight epitopes and their particular regions specific for other omptins (epitopes 1, 5–11). In fact, at least three complete epitopes (e.g., 6, 8, 10) were found to be specific for all five omptins.

### 3.3. The 3D Modeling of the Predicted Location of the Omptin Epitopes 

We visualized the location of immunoreactive clusters identified during the screening of the omptin peptide sequences ias an the 3D models of different omptins. The comparison of the ribbon diagrams of the 3D models showed high similarity between the omptin structures (Supplementary Materials Figure S1A). All four immuno-reactive regions were positioned within the surface loops. Therefore, the epitopes located in these four clusters could be the best candidates for future immunological and protective studies. The 3D models for individual epitopes of each omptin clearly demonstrated the localization of the linear (Supplementary Materials Figure S1B), conformational (Supplementary Materials Figure S1C) epitopes on the surface loops in Pla and other omptins. These favorable positions of the omptin’s epitopes make them available for direct interaction with the relevant homologous specific antibodies present in human sera and probable participation in eliciting host antibody response.

### 3.4. Omptins Allergenicity and Antigenicity Prediction 

All five omptins tested were predicted to be non-allergenic while possessing antigenic activity (Table 1). However, there were a few epitopes recognized as probable allergens (Supplementary Materials Table S2), such as epitopes 1 and 3 in OmpT, epitope 9 in OmpP, epitopes 9 and 11 in PgtE, and epitope 3 in SopA. The remaining epitopes were predicted to be non-allergenic. Nevertheless, all the omptin’s epitopes were recognized by antisera in ELISA as being antigenic and immuno-reactive. 

Finally, all five Pla-specific epitopes were predicted to be non-allergic while the other omptin-specific epitopes 1, 9, and 11 could potentially produce allergic reaction against a single omptin epitope (Supplementary Materials Table S2). Thus, the Pla epitope 2 and partial residues of the epitopes 6, 8, 10, and 11 (regions 52–60, 146–150, 231–234, 286–295, and 306–311, respectively) could be the first choice for the development of a new generation of target peptide vaccine against plague. The partial residues of omptin epitopes 6, 8, and 10 (regions 136–145, 227–230, and 274–285) could be promising targets for the multi-pathogen vaccine against a group of enterobacterial infections.

## 4. Discussion

IDs caused by omptin-expressing bacterial pathogens remain a significant cause of morbidity and mortality despite the availability of a number of licensed vaccines and many vaccine candidates under different stages of development [11,16,24,33,34]. The majority of potential vaccine candidates against enteropathogenic IDs are based on either whole-cell recombinant bacterial strains or different proteins (T3SS, T5SS, outer membrane vesicles, etc.), as well as lipopolysaccharides (LPSs) as potential immunogens. However, the wide serologic diversity of pathogens such as *Shigella* (four groups of these bacteria are pathogenic in humans, which are further divided into a total of 40 serotypes) [35], *Salmonella* (the genus includes more than 2400 serovars) [16], and *E. coli* (pathogenic bacteria are categorized into six pathotypes) [36] significantly limits the development of a universal effective mucosal enteric vaccine. Nevertheless, the main vaccine strategy is focused on the selection of promising candidate(s) with: (i) reduced LPS-associated toxicity, and (ii) ability to confer a broad serovar-independent protection against a group of the relevant IDs [16]. Here, we explored omptins representing a family of outer membrane proteases that have been identified in all major enterobacterial species pathogenic to humans [24] for the presence of similar immuno-reactive epitopes. The model pro-omptin antigen, *Y. pestis* Pla, was chosen for the following reasons: (i) the antigen is a surface-located outer-membrane integral protease with high similarity in 3D structure to other omptin family members [24]; (ii) Pla can convert plasminogen to plasmin by limited proteolysis, and this activity was likely crucial for the increased lethality of *Y. pestis* during the course of evolution [37,38,39]; (iii) Pla pro-omptin is involved in the dissemination of *Y. pestis* into circulation and is known as one of the major virulence determinants of this pathogen [40,41,42]; (iv) this protein has both species-specific and genus-specific epitopes that were detected using a panel of monoclonal antibodies [43]; (v) Pla pro-omptin activation occurs at 37 °C, coinciding with the formation of truncated less-toxic tetra-acylated LPS, which is produced instead of highly toxic hexa-acylated LPS made by *Y. pestis* at 26 °C [44]. Importantly, the Pla pro-omptin is apparently involved in pathogen–host interaction following the induction of host immune response: a detectable level of the relevant antibodies to this antigen was found in the convalescent sera of human survivors of plague infection [45], in mice that survived experimental plague [46], and in the sera of animals and humans vaccinated with live plague vaccine (LPV, EV line NIIEG) [11,28,29,30]. Finally, upon vaccination with LPV, there was a detectable level of anti-Pla secretory antibodies of IgA classes [30] suggesting the development of effective mucosal response, which is crucial in developing vaccines for both foodborne and airborne pathogens [21,47]. Importantly, immunization with purified recombinant Pla pro-omptin (i.e., the denatured inactive form of the antigen) provided no protection against plague in a murine model, although partial protection was seen in mice against the strain of *Y. pestis* lacking capsular antigen F1 [48,49]. This could indicate that both linear and conformational epitopes might be involved in eliciting protective effects. The in silico approach used in this study and data obtained by using immuno-serological investigations [30] produced nearly identical results with regard to the identification of immuno-reactive epitopes in omptins. The epitope location fell into four different extended immuno-reactive peptide clusters (Figure 1, Table 1 and Table 2) that possessed a high level of homology with other omptins (Figure 2). In fact, using the immuno-bioinformatic tools, we were able to identify several omptin peptides forming highly conserved, antigenic, predicted to be non-allergenic and immunogenic B-cell epitopes, that were least homologous with human host. Further, we narrowed down the extended epitope-containing region to a few immunogenic peptides with a high level of potency to be designed for multi-epitope vaccine candidates against either a group of enteropathogenic IDs or plague only. Moreover, we also found that human immunization with LPV elicited a long-lasting and mixed Th1/Th17 immune responses as determined with pro-omptin as the target antigen [30]. This shift towards Th17 in regularly vaccinated humans was quite possible as Th17 cell-mediated human immunity proved suppressing the IgE responses was associated with human autoimmune disease [50,51], because class switch recombination to both IgG4 and IgE depends on the production of the Th2 cytokines, particularly IL-4 and should correlate with elevated IL-10 [52]. Also, cytokine profiling revealed a slightly elevated production of IFN-γ and diminished level of IL-4 in response of peripheral blood mononuclear cells (PBMCs) to stimulation with Pla [30]. In addition, the sera of immunized donors contained a reliable level of Pla-specific immunoglobulins IgG (IgG1/IgG3) and IgA. All these observations indicate the formation of a typical Th1/Th17 immune response in humans vaccinated with LPV [30], critical for induction of both prompt and long-lasting protective clade specific, as well as serotype-independent mucosal immunity [20,21]. This was recently convincingly demonstrated by Chen K. et al. [20] on the model of *Klebsiella* pneumonia that could be approximated to other omptin-expressing pathogens. Indeed, based on a computer structural 3D model (Supplementary Materials Figure S1A–C) all immune-reactive epitopes detected by us were surface-exposed and could probably be involved in host–pathogen interaction for each omptin-expressing pathogen. This could indicate that some of these immuno-reactive epitopes (predicted or proven) may represent promising targets for the development of a new generation of effective synthetic omptin-based broad-spectrum peptide vaccine against a number of Gram-negative pathogens. Additional studies are necessary to determine individual protective characteristics of each identified epitope after experimental infection with virulent strains of homologous and heterologous enterobacterial pathogens. Moreover, it will be interesting to evaluate the comparative level of IL17A response in patients both actively infected and convalescent after IDs caused by other omptin-expressed pathogens, namely *Salmonella*, *Shigella*, and *E. coli*. On the other hand, the determination of immune correlates of human mucosal immune responses with protection is crucial for the development of new-generation vaccine candidates [47]. One of the challenges in creating universal vaccines is the poor immunogenicity of conserved antigens that was selected during evolution to avoid engaging the immune system. The proposed approach to overcome this deficiency is the structure-based engineering of immunodominant epitopes derived from different variants of the antigen to make a new immunogen capable of eliciting broad protection [53].

## 5. Conclusions

In conclusion, our data suggest excellent progress in the prompt development of a peptide-based vaccine against plague and other enteropathogenic IDs. We also suggest that it could be a single vaccine for multiple pathogens with improved characteristics due to the presence of predicted non-allergenic epitopes. These epitopes could be further evaluated in immunologic and protective studies to assess their potency as promising components of an effective broad-spectrum synthetic mucosal omptin-based peptide vaccine against omptin-expressing pathogens.

## Figures and Tables

**Table 1:** Antigenic and allergenic results of the predicted omptins ^1^.

No.	GenBank No.	Protein Name	No. of Amino Acids	No. of B-Cell Epitopes Predicted ^2^		Allergenicity	Antigenicity
				Linear	Conformational		
1	CAB53170.1	Pla	312	9	6	Non-allergen	Antigenic
2	AP001918.1	OmpP	315	11	4	Non-allergen	Antigenic
3	KU664810.1	OmpT	317	7	5	Non-allergen	Antigenic
4	ATT45876.1	PgtE	312	10	5	Non-allergen	Antigenic
5	U73461.1	SopA	315	9	4	Non-allergen	Antigenic

^1^ AlgPred [31] (http://crdd.osdd.net) and ANTIGENpro [32] (http://scratch.proteomics.ics.uci.edu/) services were used for prediction of allergenicity and antigenicity, respectively; ^2^ B-cell epitopes were predicted by ElliPro (http://tools.immuneepitope.org/toolsElliPro/).

**Table 2:** Immunopositive Pla peptides reacted with sera obtained from vaccinated (group A) and unvaccinated (group B) donors (summarized).

Peptide Immono-Reactive Cluster No.	Omptin Epitope Predicted in ElliPro	Pla Peptide Number	Positive Reaction with Sera from Donor Group		Actual Pla Peptide Position (Library Peptide ID ^1^)	Pla Peptide Position for Epitope Predicted in ElliPro
			A	B		
1	1	1–4	+	+	16–45 (4–7)	22–36
	2	5–7	+	-	41–65 (9–11)	52–60
N/A ^3^	3 ^2^	N/A	-	-	76–90 (16)	None
2	4	8–10	+	+	86–110 (18–20)	95–98
	5	11–15	+	+	101–135 (21–25)	107–124
	6	16–18	+	+	131–155 (27–29)	136–150
		19	+	-	146–160 (30)	
	7	20–24	+	+	156–190 (32–36)	163–184
3	8	26–27	+	+	221–240 (45–46)	227–234
		28	+	-	231–245 (47)	
N/A ^3^	9 ^2^	N/A	-	-	251–265 (51)	None
4	10	29–32	+	+	266–295 (54–57)	274–295
		33–34	+		286–305 (58–59)	
	11	35	+		296–310 (60)	306–312
		36		+	301–312 (61)	

^1^ described previously [30]; ^2^ unreactive peptide in Pla; ^3^ Not applicable for Pla (N/A).

## Supplementary Materials

The following are available online at https://www.mdpi.com/2076-393X/7/2/36/s1, Figure S1. Structural 3D ribbon diagrams modeling of omptin protein family based on the: (A) original amino acid sequence of Pla, OmpT, OmpP, PgtE, and SopA. The four immune-reactive clusters colored in blue (epitope no. 1), black (epitope no. 2), red (epitope no. 3), and green (epitope no. 4). All models were generated with Swiss-PdbViewer/DeepView v. 4.1. (B) B-cell linear epitopes residues of the Pla, OmpT, OmpP, PgtE, and SopA. (C) B-cell conformational epitopes residues of Pla, OmpT, OmpP, PgtE and SopA. Table S1. Predicted antigenic and allergenic results of the B-cell linear omptins.
