# Supplementary material for: New Promising Targets for Synthetic Omptin-Based Peptide Vaccine against Gram-Negative Pathogens
Source: Vaccines (Basel). 2019 Apr 10;7(2):36. doi: 10.3390/vaccines7020036 (PMC6630670; doi:10.3390/vaccines7020036)
Supplement: Supplementary file 1 [file vaccines-07-00036-s001.pdf]

vaccines

Article

New Promising Targets for Synthetic Omptin-Based Peptide Vaccine against Gram-Negative Pathogens

Valentina A. Feodorova [1,*], Anna M. Lyapina [1], Sergey S. Zaitsev [1], Maria A. Khizhnyakova [1], Lidiya V. Sayapina [2], Onega V. Ulianova [1], Sergey S. Ulyanov [3] and Vladimir L. Motin [4,*]

Supplementary Materials:

A

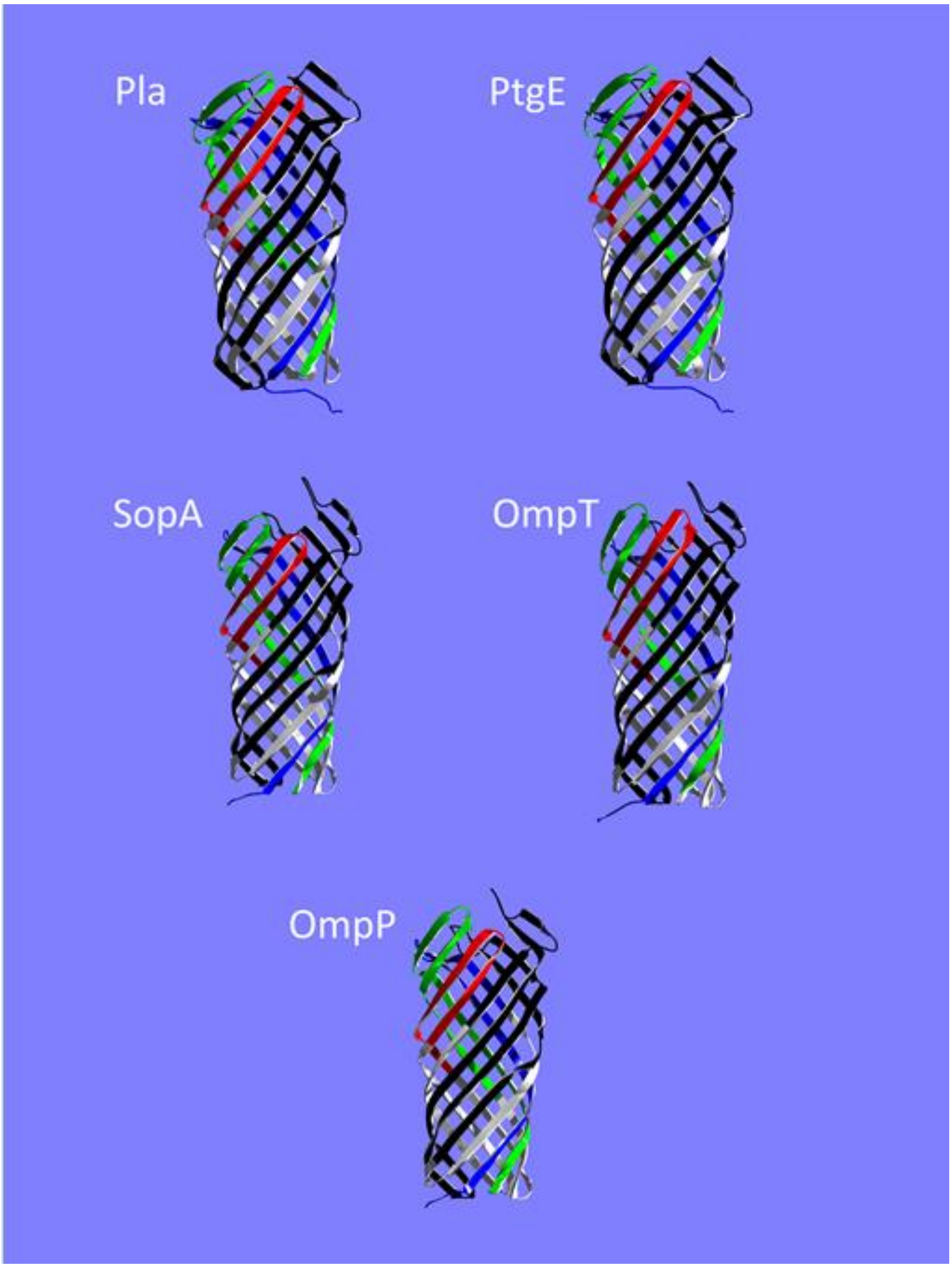

B

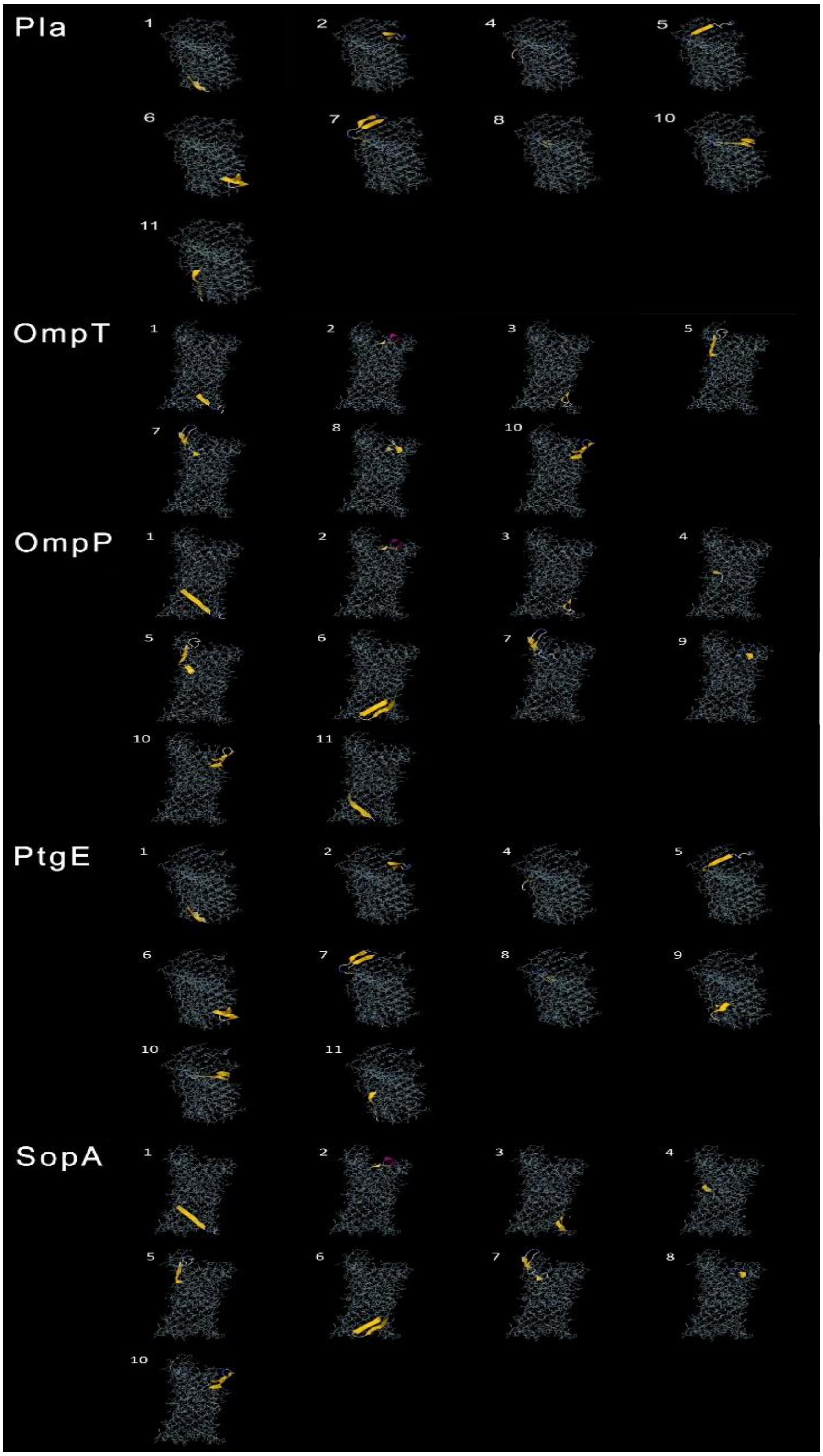

Pla
1
2
4
5
6
7
8
10
11
OmpT
1
2
3
5
7
8
10
OmpP
1
2
3
4
5
6
7
9
10
11
PtgE
1
2
4
5
6
7
8
9
10
11
SopA
1
2
3
4
5
6
7
8
10

C

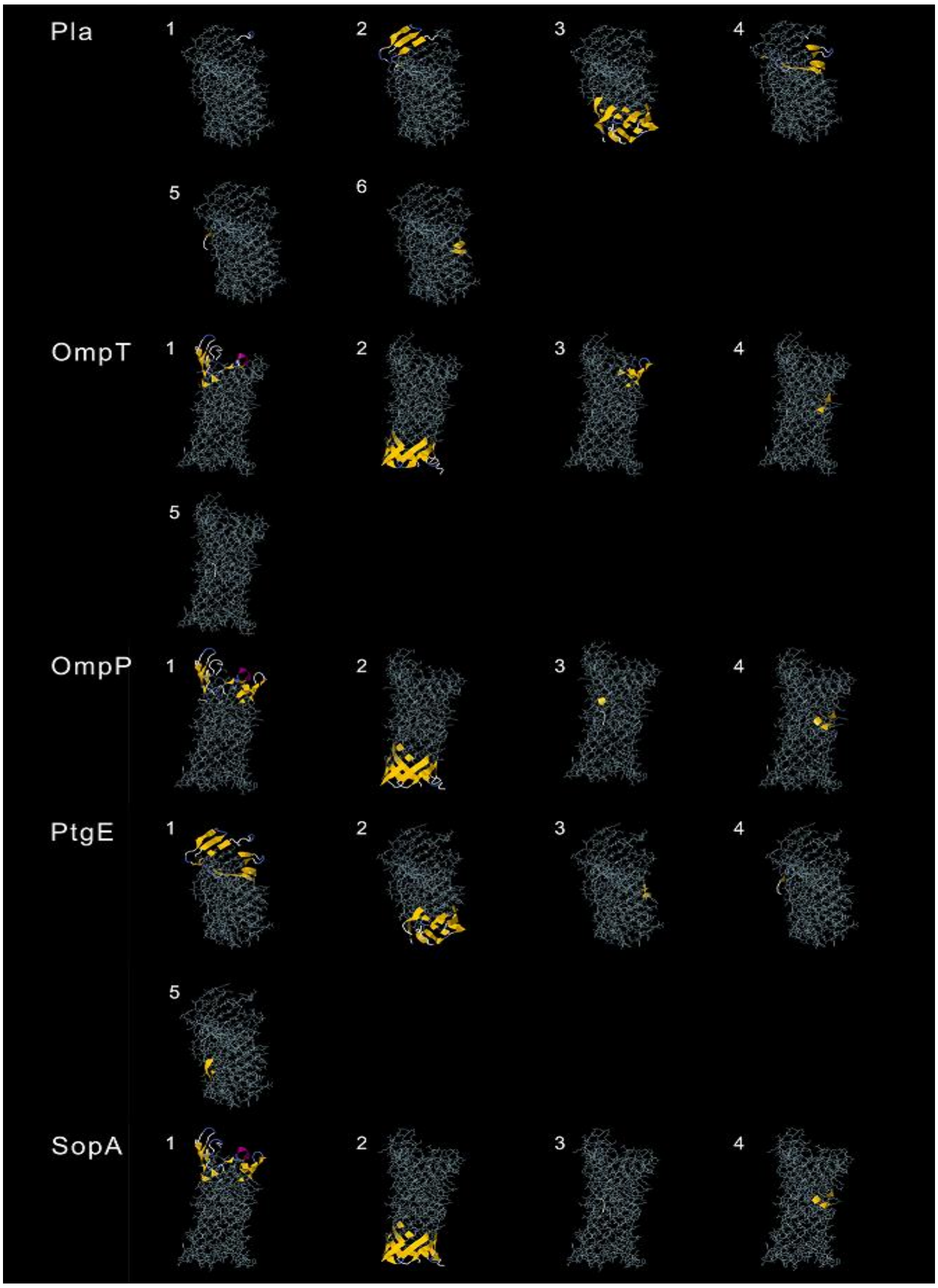

Figure S1. Structural 3D ribbon diagrams modeling of omptin protein family based on the: (**A**) original amino acid sequence of Pla, OmpT, OmpP, PgtE, and SopA. The four immune-reactive clusters colored in blue (epitope no. 1), black (epitope no. 2), red (epitope no. 3), and green (epitope no. 4). All models were generated with Swiss-PdbViewer/DeepView v. 4.1. (**B**) B-cell linear epitope residues of the Pla, OmpT, OmpP, PgtE, and SopA. (**C**) B-cell conformational epitope residues of Pla, OmpT, OmpP, PgtE, and SopA.

Table 1S. Predicted antigenic and allergenic results of the B-cell linear omptins.

Epitope No.	Omptin	Epitope Sequence[2]	Allergenicity of the B-Cell Epitope
1	Pla	SSQLIPNISPDSFTV	Non-Allergenic
	PgtE	SALFIPDVSPDSVTT	Non-Allergenic
	SopA	FIPDNISTDISL	Non-Allergenic
	OmpT	LSFTPEKIST	Probable Allergen
	OmpP	FGPEKISTEINL	Non-Allergenic
2	Pla	*YDAETGRKI*	Non-Allergenic
	PgtE	YDTDTGRKL	Non-Allergenic

	SopA	HPKEGGR	Non-Allergenic
	OmpT	LPEEKGR	Non-Allergenic
	OmpP	EPEEGGR	Non-Allergenic
	Pla	-	-
	PgtE	-	-
3	SopA	WKLIPKVSF	Probable Allergen
	OmpT	WDLLPRVSV	Probable Allergen
	OmpP	WELNPWLSV	Non-Allergenic
	Pla	LASG	Non-Allergenic
	PgtE	LASG	Non-Allergenic
4	SopA	LGNQK	Non-Allergenic
	OmpT	-	-
	OmpP	LNSR	Non-Allergenic
	Pla	WMNENQSEWTDHSSHPAT	Non-Allergenic
	PgtE	WMSSEQPGWTDRSIHPD	Non-Allergenic
5	SopA	WNNSNTPQVWTDQSWHPN	Non-Allergenic
	OmpT	WLDTSNPGTWTDESKHPN	Non-Allergenic
	OmpP	WMDSGTPGTWTDESRHPDTR	Non-Allergenic
	Pla	VKGWLLQDEN*YKAGI*	Non-Allergenic
	PgtE	VKGWLLQGDNYKAGV	Non-Allergenic
6	SopA	LKGWLLNNLDYRLGL	Non-Allergenic
	OmpT	-	-
	OmpP	VKGWFLKESDYRLAI	Non-Allergenic
	Pla	ATGGSYSYNNGAYTGNFPKGVR	Non-Allergenic
	PgtE	RGGSYIYDNGRYIGNFPHGVR	Non-Allergenic
7	SopA	MGGSYIYSENGGSRNKKGAHPSGERT	Non-Allergenic
	OmpT	KGGSYIYSSEGGFRDETGSFPDGER	Non-Allergenic
	OmpP	TGGTYIYSENGGFRNETGALPDKIKV	Non-Allergenic
	Pla	EHYM*RDLT*	Non-Allergenic
	PgtE	EHYMRKLT	Non-Allergenic
8	SopA	YQTETI	Non-Allergenic
	OmpT	YNPEKRIT	Non-Allergenic
	OmpP	YVRQTT	Non-Allergenic
	Pla	-	-
	PgtE	GYYITSNAKI	Probable Allergen
9	SopA	-	
	OmpT	-	
	OmpP	AGYYITPEAKVYI	Probable Allergen
	Pla	KGGTQTIDKNSG*DSVSIGGDAA*	Non-Allergenic
	PgtE	KGGTQIIDKTSGDTAYFGGDA	Non-Allergenic
10	SopA	KGDTSLYEQSTNISGTIKNS	Non-Allergenic
	OmpT	KGDTSLYSRNLNISDHTKNG	Non-Allergenic
	OmpP	KGDTSLYDRSDNTSEHNNNG	Non-Allergenic
	Pla	*AGLQYRF*	Non-Allergenic
	PgtE	*AGLQYRF*	Probable Allergen
11	SopA	-	-
	OmpT	-	-
	OmpP	GTALKYTF	Non-allergenic

[1] AlgPred[31] (http://crdd.osdd.net/raghava/algpred/index.html) and AllerTOP[32] v. 2.0 (http://www.ddg-pharmfac.net/AllerTOP) services were used for prediction of allergenicity. [2] B-cell epitopes were predicted by ElliPro (http://tools.immuneepitope.org/toolsElliPro/).

© 2019 by the authors. Submitted for possible open access publication under the terms and conditions of the Creative Commons Attribution (CC BY) license (http://creativecommons.org/licenses/by/4.0/).
